# Ultrasound-targeted microbubble destruction promotes PDGF-primed bone mesenchymal stem cell transplantation for myocardial protection in acute Myocardial Infarction in rats

**DOI:** 10.1186/s12951-023-02204-7

**Published:** 2023-12-15

**Authors:** Zhenxing Sun, Yu Cai, Yihan Chen, Qiaofeng Jin, Ziming Zhang, Li Zhang, Yuman Li, Lei Huang, Jing Wang, Yali Yang, Qing Lv, Zhengyang Han, Mingxing Xie, Xiangming Zhu

**Affiliations:** 1 Anhui Medical University, Hefei, 230031 China; 2 Present Address: Department of Ultrasound, The First Affiliated Hospital of Wannan Medical College, Wuhu, 241001 China; 3 Department of Ultrasound Medicine, Union Hospital, Tongji Medical College, Huazhong University of Science and Technology, Wuhan, 430022 China; 4 Hubei Province Key Laboratory of Molecular Imaging, 430022 Wuhan, China; 5 Department of Ultrasound, The First Affiliated Hospital of Zhengzhou University, Zhengzhou, 450052 China

**Keywords:** Ultrasound-targeted microbubble destruction, Bone mesenchymal stem cells, Acute Myocardial Infarction, PDGF-BB

## Abstract

**Background:**

Ultrasound-targeted microbubble destruction (UTMD) has emerged as a promising strategy for the targeted delivery of bone marrow mesenchymal stem cells (MSCs) to the ischemic myocardium. However, the limited migration capacity and poor survival of MSCs remains a major therapeutic barrier. The present study was performed to investigate the synergistic effect of UTMD with platelet-derived growth factor BB (PDGF-BB) on the homing of MSCs for acute myocardial infarction (AMI).

**Methods:**

MSCs from male donor rats were treated with PDGF-BB, and a novel microbubble formulation was prepared using a thin-film hydration method. In vivo, MSCs with or without PDGF-BB pretreatment were transplanted by UTMD after inducing AMI in experimental rats. The therapeutic efficacy of PDGF-BB-primed MSCs on myocardial apoptosis, angiogenesis, cardiac function and scar repair was estimated. The effects and molecular mechanisms of PDGF-BB on MSC migration and survival were explored in vitro.

**Results:**

The results showed that the biological effects of UTMD increased the local levels of stromal-derived factor-1 (SDF-1), which promoted the migration of transplanted MSCs to the ischemic region. Compared with UTMD alone, UTMD combined with PDGF-BB pretreatment significantly increased the cardiac homing of MSCs, which subsequently reduced myocardial apoptosis, promoted neovascularization and tissue repair, and increased cardiac function 30 days after MI. The vitro results demonstrated that PDGF-BB enhanced MSC migration and protected these cells from H_2_O_2_-induced apoptosis. Mechanistically, PDGF-BB pretreatment promoted MSC migration and inhibited H_2_O_2_-induced MSC apoptosis via activation of the phosphatidylinositol 3-kinase/serine-threonine kinase (PI3K/Akt) pathway. Furthermore, crosstalk between PDGF-BB and stromal-derived factor-1/chemokine receptor 4 (SDF-1/CXCR4) is involved in the PI3K/AKT signaling pathway.

**Conclusion:**

The present study demonstrated that UTMD combined with PDGF-BB treatment could enhance the homing ability of MSCs, thus alleviating AMI in rats. Therefore, UTMD combined with PDGF-BB pretreatment may offer exciting therapeutic opportunities for strengthening MSC therapy in ischemic diseases.

**Supplementary Information:**

The online version contains supplementary material available at 10.1186/s12951-023-02204-7.

## Introduction

Acute myocardial infarction (AMI) remains a leading cause of morbidity and mortality worldwide [1, 2]. Severe ischemia causes extensive cardiomyocyte loss and myocardial fibrosis leading to cardiac remodeling and heart failure that cannot be reversed. Bone marrow mesenchymal stem cells (MSCs) have been proposed as a promising therapy for the repair of ischemic myocardium by releasing paracrine factors into the surrounding tissue, which prevents heart failure development after AMI [3, 4]. However, the insufficient homing of intravenously injected stem cells at the site of myocardial ischemia remains a major problem [5, 6]. Therefore, the improvement of stem cell homing is a constant challenge in the field of stem cell transplantation.

Numerous studies have demonstrated that ultrasound-mediated microbubble destruction (UTMD) serves as an efficient method for the targeted delivery of stem cells following myocardial infarction (MI) by enhancing the permeability of the cell membrane, rupturing the microvascular wall, expanding the gap between endothelial cells and changing the local myocardial microenvironment [7, 8]. In addition, UTMD facilitates the homing of MSCs into the myocardial infarction zone without inducing adverse effects on the proliferation, apoptosis and cell cycle of the transplanted stem cells. However, MSC transplantation relying only on UTMD has limitations because chemokine receptor 4 (CXCR4) expression is markedly reduced during ex vivo expansion of the cells, leading to decreased migration toward the stromal-derived factor-1 (SDF-1) gradient [9, 10]. Therefore, strategies to mobilize the internalized receptor and increase CXCR4 expression are critical for improving the migration of MSCs.

Platelet-derived growth factor (PDGF) comprises homodimers or heterodimers of A- and B-polypeptide chains that exert their biological effects by binding to two structurally related tyrosine kinase receptors, the PDGF-α and PDGF-β receptors [11, 12]. PDGF-BB and PDGFR-β interaction induces the phosphorylation of PDGFR and activates the phosphatidylinositol 3-kinase/serine-threonine kinase (PI3K/AKT) signaling pathway, which contributes to numerous cell processes, including cell proliferation, survival, motility, and angiogenesis [12, 13]. A previous study demonstrated that PDGF-BB enhanced CXCR4 expression through PDGFR-β in a dose- and time-dependent manner [14]. As a vital member of the PDGF family, PDGF-BB plays a crucial role in the migration, survival, and anti-apoptosis of MSCs [15–18]. However, whether PDGF-BB enhances the migration and antiapoptotic ability of MSCs via the upregulation of CXCR4 and whether crosstalk between PDGF-BB/PDGFR-β and SDF-1/CXCR4 is involved in the PI3K/AKT signaling pathway remains unknown. Thus, it is necessary to identify whether these signaling molecules participate in signal transduction in the migration and anti-apoptosis of MSCs and CXCR4 expression regulation induced by PDGF-BB.

**Fig. 1:**
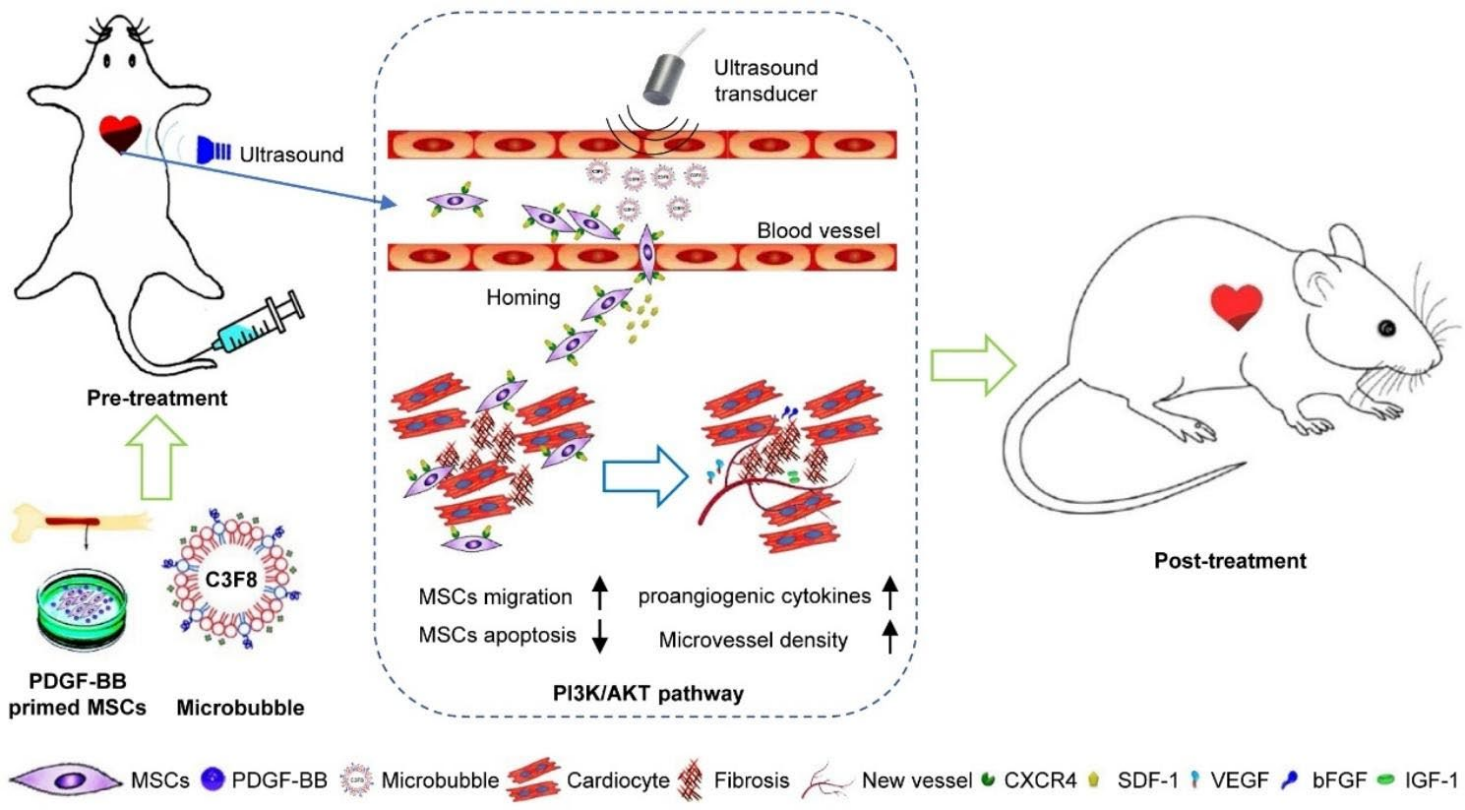
Schematic representation of PDGF-BB-primed MSC transplantation by UTMD for infarcted myocardium repair. UTMD enhanced the delivery of MSCs into the infarcted myocardium by upregulating SDF-1 expression. Compared with UTMD alone, UTMD combined with PDGF-BB pretreatment increased the therapeutic effect of grafted cells by improving MSC migration and survival, reducing cardiomyocyte apoptosis, decreasing fibrosis, increasing microvessel density (via upregulation of VEGF, bFGF and IGF-1) and improving cardiac function. PDGF-BB promotes the survival/retention and cardioprotection of engrafted MSCs in rat models of MI via the PI3K/Akt pathway and CXCR4 activation

In this study, we hypothesized that UTMD combined with PDGF-BB pretreatment could increase the therapeutic effect of grafted cells in a rat model of MI and that the PI3K/Akt signaling pathway plays a role in the PDGF-BB-induced cell migration and apoptosis resistance of MSCs. Thus, the present study aimed to investigate whether UTMD combined with PDGF-BB pretreatment further increased the therapeutic effect of engrafted MSCs in rat models of MI. Furthermore, the underlying cellular and molecular mechanisms involved in the cardioprotection of PDGF-pretreated MSCs were also examined. We demonstrated evidence that transplantation of PDGF-BB-primed MSCs by UTMD improves cardiac function by reversing myocardial remodeling and reducing the size of infarction in a rat model of AMI. Furthermore, our results revealed that PDGF-BB promotes the survival/retention and cardioprotection of engrafted MSCs in rat models of MI via the PI3K/Akt pathway and CXCR4 activation. We therefore conclude that this minimally invasive stem cell transplantation strategy combined with PDGF-BB pretreatment may offer exciting therapeutic opportunities for strengthening MSC therapy in AMI. A schematic representation of the UTMD-mediated PDGF-BB-primed MSC therapeutic strategy is shown in Fig. 1.

## Materials and methods

### Animals

Sprague‒Dawley (SD) rats were used in this study. MSCs were obtained from male SD rats (60–80 g), and female SD rats were used for AMI modeling (200–220 g). The rats were housed in an SPF animal room at a temperature of 22 ~ 25 °C and humidity of 55 ~ 60% with a 12 h/h dark/light cycle. All animals received humane care, and the experimental protocol was approved by the Huazhong University of Science and Technology Animal Care and Use Committee.

### Preparation of the CMBs

The cationic microbubbles (CMB) coated with 1,2-distearoyl-sn-glycerin-3-phosphate choline (DSPC), 1,2-distearoyl-sn-glycerin-3- phosphate ethanolamine-n-[methoxy (polyethylene glycol)-2000] (DSPE-PEG2000) and 3-[N-(N’, N’-dimethyl-lamino-ethane)-carbamoyl] cholesterol (DC-CHOL) (Avanti Polar Lipids Inc., Alabaster, AL, USA) with an octafluoropropane (C3F8) gas core (Foshan Huate Gas Co. Ltd., Foshan, China) were prepared by the thin-film hydration and sonication method as described in our previous study [19]. Briefly,

DPPC, DC-CHOL and DSPE-PEG2000 were weighed and dissolved in a flask with chloroform at a molar ratio of 10:4:1. A lipid film was formed by removing the organic solvent from the phospholipid solution with a rotary evaporator under high vacuum pressure. The mixture was incubated under vacuum and dry conditions overnight. Phosphate-buffered saline (PBS) was added, and the mixture was then hydrated on a rotary evaporator. The mixture was sonicated with octafluoropropane (C3F8) gas to prepare the CMB suspension. The C3F8 gas was bubbled through the solution for 1 min. The mixture was then sonicated (20 kHz, 33–42 W). The lipid shell of the neutral microbubbles (NMBs) in this study consisted of DPPC and DSPE-PEG2000 at a molar ratio of 5:2. After preparation, PBS was used to adjust the concentration of the microbubbles to 1 × 10^9^ microbubbles/ml. Subsequently, the MB solution was sterilized by ^60^Co-γ radiation and stored at 4 °C until further use.

### Characterization of the CMBs

A light microscopy image of the CMBs was obtained with an optical microscope (model IX70, Olympus Inc., Melville, NY, USA). The surface morphologic characteristics of the CMBs were determined by transmission electron microscopy (TEM, Hitachi H-7000FA, Japan). The size distribution, concentration and zeta potential of the CMBs were measured using a Zetasizer NANO ZS system (Malvern Instruments Ltd., Malvern, UK). Bubble stability in solution was analyzed and measured, and the imaging ability of the CMBs at different concentrations was assessed in vitro with a homemade agarose phantom. The imaging properties of the CMBs for rat hearts were evaluated in vivo. Images were acquired and analyzed using a clinical IU22 ultrasound scanner (Philips Medical Systems, Amsterdam, Netherlands).

### Cell culture and conditioned medium collection

MSCs from the femoral and tibial bone marrow of SD rats were isolated and cultured as previously described [20]. Briefly, 3-week-old healthy male rats (60–80 g) were anesthetized (1% pentobarbital sodium, 40 mg/kg) and sacrificed by cervical dislocation. The bone marrow cavities of the tibia and femur were flushed with serum-free Dulbecco’s Modified Eagle Medium (DMEM) with F12 (Gibco, Grand Island, NY, USA), and the flushing fluid was collected. After centrifugation, the cells suspended in DMEM/F12 medium containing streptomycin (100 g/mL), penicillin (100 U/mL) and 10% FBS (fetal bovine serum; Gibco) were cultured at 37 °C in wet air containing 5% CO2. The medium was changed every two days to remove nonadherent cells. MSCs were observed using an optical microscope (model IX70, Olympus Inc., Melville, NY, USA). The phenotypic properties of MSCs were identified by flow cytometric analysis for the expression of the typical markers CD90 and CD29 (BD Biosciences) and the absence of the hematopoietic markers CD45 (eBioscience) and CD34 (Santa Cruz Biotechnology). MSCs at passages 3 and 4 were used for the subsequent experiments. The cells were seeded at 5 × 10^5^ cells per 10-cm plate to prepare conditioned medium (CM) from MSCs. When the MSCs reached 80% confluence, they were placed in serum-free medium for 24 h. The CM was then collected for in vitro experiments. Similarly, MSCs were cultured with H_2_O_2_ for 6 h before MSC-CM use.

### Cell migration assay

The effect of PDGF-BB on MSC migration was examined using Transwell cell migration assay and in vitro scratch wound-healing assays. MSCs were seeded in 6-well plates. When the cells reached 90% confluence, MSCs were scratched with a 200-µl pipette and washed with PBS. MSCs were incubated with or without PDGF-BB at different concentrations (R&D Systems) in serum-free DMEM/F12 for 24 h. Images of the scratched gaps were captured at 0 and 24 h with an inverted microscope (IX70, Olympus Inc., Melville, NY, USA), and the cell migration area was measured using ImageJ software (NIH, USA).

Next, MSCs were subjected to Transwell assays as described previously [21]. Briefly, MSCs (1 × 10^5^ cells per well) were treated with PDGF-BB at the optimum concentration (R&D Systems) or vehicle and were then resuspended in DMEM/F12 with 1% FBS and seeded into the upper chamber of the Transwell system (8.0 μm pore size, Merck, USA). DMEM/F12 containing 10% fetal bovine serum (500 µL) with or without 100 ng/ml SDF-1 (PeproTech, USA) was added to the lower chamber. After 24 h of culture in the cell incubator, the noninvading cells on the upper surface were swabbed with a cotton swab. The inserts were fixed in 4% paraformaldehyde at room temperature for 30 min and stained with crystal violet for 20 min. The number of MSCs across the membrane was calculated in five randomly selected regions under an optical microscope (IX70, Olympus Inc., Melville, NY, USA). In some experiments, a suspension of PDGF-BB-primed MSCs containing 50 µM LY294002 (a PI3K inhibitor, Abcam) or 44 nM AMD3100 (a CXCR4 inhibitor, Abcam) was added to the upper chamber to neutralize PI3K/Akt and CXCR4 bioactivity, respectively.

### Cell apoptosis analysis by flow cytometry

Cell apoptosis was analyzed using an Annexin V-FITC Apoptosis Detection Kit (eBioscience) following the manufacturer’s instructions. Briefly, the cells were collected, washed with PBS and resuspended in 200 µl binding buffer. Subsequently, the cells were mixed with 5 µl Annexin V-FITC at room temperature for 10 min following incubation with 10 µl propidium iodide (PI). Finally, early and late apoptosis were analyzed using flow-assisted cell sorting (FACS) (FACS Calibur, BD).

### Rat model of acute Myocardial Infarction

The AMI model was established in female SD rats as previously described [22, 23]. Briefly, rats (200–220 g) were anesthetized with 1% pentobarbital sodium (40 mg/kg, administered intraperitoneally), intubated and ventilated. Then, the left thoracic cavity was opened, and the left anterior descending (LAD) coronary artery was ligated 2–3 mm from the tip of the left auricle with a 6/0 suture to trigger AMI. Successful ligation of the LAD was confirmed by the appearance of a Q wave and S-T segment elevation on an electrocardiogram. Sham-operated control rats underwent the same surgical procedures except that the suture placed under the left coronary artery was not tied.

### Cell labeling

MSCs were labeled with DiR (Caliper Life Sciences, Hopkinton, MA, USA) or green GFP using a lentiviral vector (Genechem Ltd, Shanghai, China) following the manufacturer’s instructions to enable cell tracing after transplantation in vivo.

### MSC delivery by UTMD

The distribution and homing of MSCs after UTMD-mediated delivery were then evaluated. SD rats were randomly subjected to the following four treatments: Sham, PBS (1 mL) infusion only; MI, PBS (1 mL) infusion only; MI-MSC, 2 × 10^6^ DiR- or GFP-labeled MSCs suspended in 1 mL of PBS infusion; and MI-MSC-UTMD, 2 × 10^6^ DiR- or GFP-labeled MSCs suspended in 1 mL of PBS infusion. SD rats were subjected to MI and then underwent UTMD followed by intravenous injection of MSCs. MSCs were labeled with DiR or GFP using a lentivirus before targeted delivery mediated by UTMD. Rats in the MI-MSC-UTMD group underwent UTMD followed by cell transplantation. Rats in the other groups did not receive UTMD.

The effects of UTMD combined with PDGF-BB pretreatment on MSC therapy were also evaluated in the rat MI model. SD rats were divided into four groups: (i) sham, PBS (1 mL) infusion only; (ii) MI, PBS (1 mL) infusion only; (iii) MI-MSC-vehicle, 2 × 10^6^ DiR- or GFP-labeled MSCs suspended in 1 mL of PBS infusion; and (iv) MI-PDGF-BB-MSC, 2 × 10^6^ DiR- or GFP-labeled MSCs suspended in 1 mL of PBS infusion. MSCs were pretreated with PDGF-BB (50 ng/ml) for 24 h and labeled with GFP using a lentivirus before delivery via UTMD.

Rats in the groups treated with MSCs underwent UTMD followed by cell transplantation. CMBs (120 µl/rat) were subsequently diluted in saline to a total volume of 500 µl/rat. A micropump was used to infuse 0.5 mL of CMBs at a rate of 15 ml/h during ultrasound irradiation. UTMD was performed using a therapeutic ultrasound system (Sonitron 2000 V, Japan) at 1 MHz, 20% duty ratio and 2.0 W/cm^2^ output intensity for two minutes directed to the anterior left ventricular wall. The acoustic window for each rat was confirmed by a diagnostic ultrasound system (IU22, Philips, Bothell, MA, USA). After UTMD, 2 × 10^6^ DiR- or GFP-labeled MSCs with or without PDGF-BB pretreatment were injected through the caudal vein. Rats in the other groups without MSC treatment also received UTMD prior to the 1 mL PBS injection. MSC transplantation mediated by UTMD was performed at 2-day intervals between Day 1 and Day 5 post-MI. A dose of furosemide (0.4 mg/kg) was injected before MSC transplantation to prevent congestive heart failure due to volume overload.

### Ex vivo bioluminescent imaging

The histological distribution of DiR-labeled MSCs was monitored using bioluminescent imaging (BLI) at 3 days after MI. All rats used for ex vivo imaging, were fed an alfalfa-free diet to reduce fluorescent noise. DiR-labeled MSCs were intravenously injected into MI rats following UTMD. At 3 days post-MI, three rats from each group were sacrificed, and the major organs were harvested, including the hearts, livers, spleens, lungs and kidneys. Then, ex vivo images of the organs were captured and quantified using a small animal imaging system (In-Vivo FX PRO, Bruker, USA) to visualize cell homing and distribution.

### Detection and tracking of GFP-labeled MSCs

A multimodal evaluation strategy was applied to assess the retention of GFP-labeled MSCs comprising flow cytometry, qPCR and immunofluorescence staining. The flow cytometry assay was conducted following the transplantation of MSCs. The heart samples were finely minced, and a cell suspension was obtained by digestion with a solution of 1 mg/ml collagenase/dispase and 200 µg/ml DNase in alpha-MEM containing 5% FBS at 37°C with constant agitation. Following digestion, the cells were washed, and GFP-labeled cells were quantified using a Novocyte Flow Cytometer (ACEA Bioscience, USA). The percentage of positive cells was measured, and the number of positive cells was normalized to the total number of cells in the sample. To validate the Novocyte Flow Cytometer-based findings, quantitative real-time PCR of the Y-chromosome Sry-gene in infarcted myocardium was performed as previously described [24]. Briefly, tissues were processed, nucleic acids were extracted, and gDNA concentrations and purities were measured by UV absorbance. The male rat Sry gene sequence was detected on the background of female rat gDNA using a TaqMan PCR kit (Applied Biosystems). The target gene primer sequences were 5’-CATCGAAGGGTTAAAGTGCCA-3’ and 5’-ATAG TGTGTAGGTTGTTGTCC-3’. The survival rate of transplanted cells in each group was calculated as previously described [24]. In addition, immunofluorescence staining was performed to evaluate GFP-positive MSCs in the heart. The hearts were harvested and rapidly frozen in liquid nitrogen. Serial 5 μm sections were prepared, stained with a-actin (Sigma, USA) and DAPI (Sigma, USA), and observed under a laser scanning confocal microscope (model IX70, Olympus Inc., Melville, NY, USA). The numbers of GFP-positive cells on each slide were analyzed using ImageJ software (NIH, USA).

### Echocardiography

Transthoracic echocardiography was performed to evaluate left ventricular function before and 30 days after MI. A commercially available echocardiographic system (GE, VIVID7, USA) outfitting a 10 MHz transducer was employed by an investigator blinded to group designation.

Briefly, rats from different treatment groups were anesthetized with 1% pentobarbital sodium (40 mg/kg) and placed on the experimental platform. Two-dimensional guided M-mode tracings were recorded from the parasternal long-axis view of the left ventricle. The left ventricular ejection fraction (LVEF) and left ventricular fractional shortening (LVFS) of the rats were measured. Variations in the LVEF and LVFS were calculated by subtracting the values at endpoints from baseline values. Dimension data are presented as the average measurements of three cardiac cycles.

### Masson staining

Thirty days after MI, rats from different treatment groups were anesthetized and perfused with normal saline. The heart of each rat was removed quickly, sectioned and fixed in 4% paraformaldehyde solution. Paraffin-embedded slides were stained with Masson’s trichrome. Scar size (%) was measured as the percentage of fibrotic area in the total left ventricular area. The figure (Masson trichrome stain, original magnification ×200) was processed with ImageJ software to calculate the percent fibrosis in the peri-MI area. The thickness (µm) of the infarcted cardiac wall was calculated as the mean of three equidistant measurements on each section. Fluorescence microscopy (model IX70, Olympus Inc., Melville, NY, USA) was used to capture images, and ImageJ software (NIH, USA) was used for the analysis.

### Western blotting

Total proteins were extracted from MSCs or myocardial infarct tissue with radioimmunoprecipitation assay lysis (RIPA) buffer containing protease and phosphatase inhibitors for Western blotting as previously described [25]. The concentrations of proteins were determined with a Bradford protein assay kit (Bio-Rad, Richmond, CA, USA) using BSA as a protein standard. Proteins were separated by SDS 10% PAGE and transferred to polyvinylidene fluoride membranes (Amersham Biosciences, GE Healthcare, France). The membranes were blocked in 3% milk for 1 h and then incubated with primary antibody at 4 °C overnight. Following primary antibodies were utilized in this study: PI3K (#AF6241, Affinity), p-PI3K (#AF3242, Affinity), AKT (#10176-2-AP, proteintech), p-AKT (#AF0016, Affinity), ERK (#11257-1-AP, proteintech), p-ERK (#28733-1-AP, proteintech), CXCR4 (#AF5279, Affinity), Caspase 3 (#9662, Cell Signaling), cleaved-Caspase 3 (#9664, Cell Signaling), Bax (#BA0315-2, BOSTER), BCL-2 ((#MA00040, BOSTER), VEGF (#BA0407, BOSTER), bFGF (#CY3239, Abways), and IGF-1 (ab182408, Abcam) with GAPDH (#GB11002, Servicebio) as internal reference. HRP-linked rabbit IgG (#G1213, Servicebio) was used as a secondary antibody. The density of the respective bands was quantified using a densitometer with Quantity One software (Bio-Rad).

### TUNEL staining

A TUNEL apoptosis kit (Roche Applied Science, South San Francisco, CA, USA) was used to determine cardiomyocyte apoptosis according to the manufacturer’s directions. Cardiomyocytes were stained with cardiac troponin I (Abcam), and nuclei were stained with DAPI (Sigma Aldrich, USA). Sections were imaged using a confocal microscope (IX70, Olympus Inc. Melville, NY, USA). The total number of nuclei and TUNEL-positive nuclei were quantitated using Image-Pro Plus analysis software. The average number of positive cells per high-power field was calculated to assess cardiomyocyte apoptosis.

### Immunofluorescence staining

Immunofluorescence staining of tissue sections was performed as previously described [26]. Briefly, heart tissues were collected, fixed with 4% PFA, embedded in paraffin, and sectioned. For the immunofluorescence analyses, heart sections were stained with primary antibodies against α-SMA (Abcam) and CD31 (BD Bioscience). DAPI was used for nuclear counterstaining. Images were obtained with a fluorescence microscope (IX70, Olympus Inc. Melville, NY, USA). Quantification of all data was performed using Image-Plus image analysis software.

### Statistical analysis

All experiments were performed at least three times, and representative results are shown. All results are summarized as the mean ± standard error (M ± SEM). Independent two-sample t tests or one-way ANOVA were used to evaluate the significance of the differences between different groups. The data were analyzed using SPSS 26.0 software. P < 0.05 was considered to indicate significance.

## Results

### Characterization of the CMBs

**Fig. 2:**
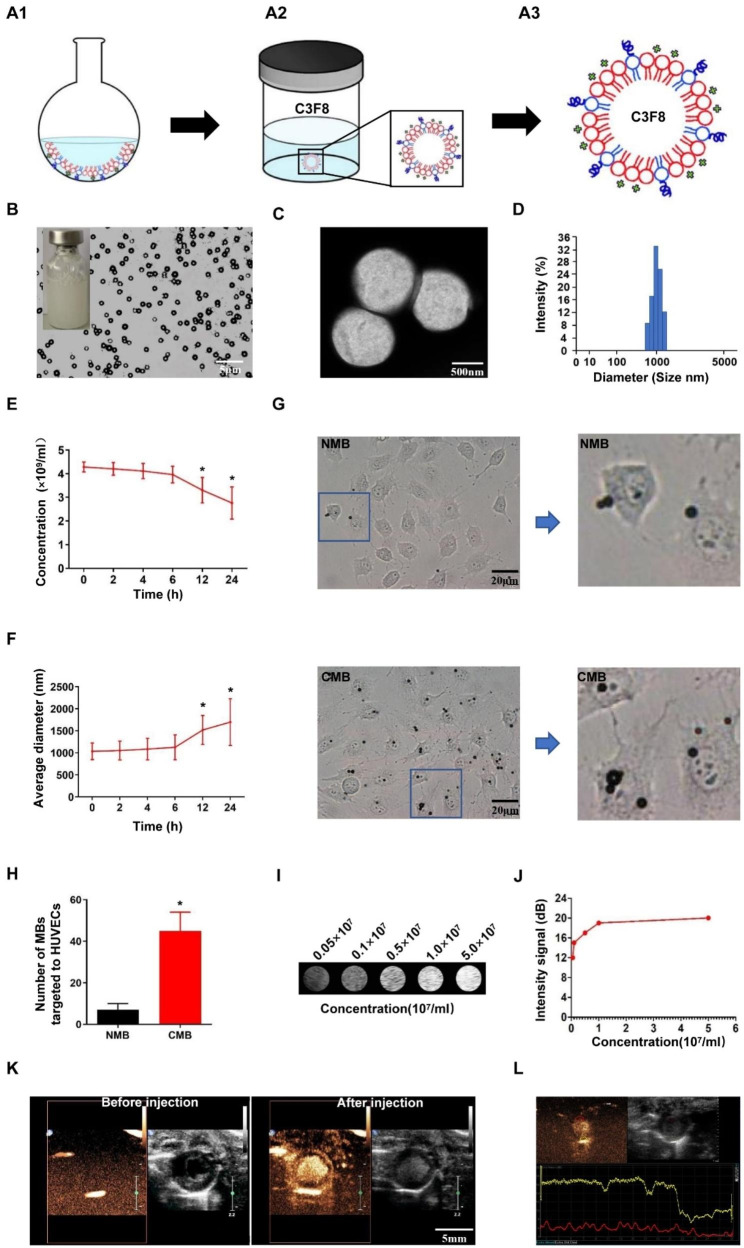
Fabrication and characterization of CMB (**A**) Schematic illustration of the fabrication process of CMBs. (**B**-**C**) Morphology of CMBs observed by optical microscopy (bar, 50 μm) and TEM (bar, 500 nm), showing that the CMBs were spherized, uniform in size and well distributed. (**D**) Size distribution of CMBs. (**E**-**F**) Changes in the concentration and mean diameter of CMBs at different time points. Values are the mean ± SEM. Significant differences were determined by Student’s t test. N = 5/group. * P < 0.05 vs. 0 h. (**G**) NMB and CMB targeted to HUVECs observed under an optical microscope. Bar, 20 μm. (**H**) Quantitative evaluation of microbubbles targeted to HUVECs using Image Plus software. Values are the mean ± SEM. Significant differences were determined by Student’s t test. N = 5/group. * P < 0.05 vs. NMB. (**I**) In vitro imaging of MBs at different concentrations. (J) Quantitative analysis showed that the contrast signal increased with increasing MB concentration. (K-L) In vivo imaging of the heart to assess infarcted myocardial perfusion and the imaging capability of CMBs. Bar, 5 mm

CMB fabrication is illustrated in Fig. 2A (see Methods for more details). The CMBs appeared as a transparent liquid that turned milky-white after oscillation (Fig. 2B). The CMBs appeared as a bright gas core surrounded by dark circular rings under the inverted microscope and regular spherical particles on SEM (Fig. 2B-C). The CMB concentration was (4.28 ± 0.21) ×10^9^/ml. The synthesized CMBs had a zeta potential of 27.09 ± 3.09 mV and a mean diameter of 1036 ± 191 nm. Figure 2D shows the CMB size distribution. The CMB properties are listed in Table 1. The CMBs had prominent stability in concentration and diameter 6 h after preparation. Then, the CMB concentration decreased slightly at 12 h (P<0.05 vs. 0 h) and significantly at 24 h (P < 0.01 vs. 0 h, Fig. 2E). In addition, the average diameter of the CMBs increased slightly at 12 h (P <0.05 vs. 0 h) and significantly at 24 h (P < 0.01 vs. 0 h, Fig. 2F). The attachment of CMBs to HUVECs was obviously higher than that of NMBs (P <0.05 vs. NMB, Fig. 2G-H). Moreover, the contrast imaging capability of CMBs was tested. The in vitro imaging results revealed that the CMB signal enhanced as the concentration increased (Fig. 2I-J). As shown in Fig. 2K, no ultrasound imaging signals were observed in B-mode before the injection of CMBs, whereas these signals were obvious after the injection. After enhanced ultrasonography, the digital images were analyzed by QLAB software (Philips Medical Systems HSG) to assess myocardial perfusion (Fig. 2L). The results revealed the high stability and excellent imaging features of CMBs, which is crucial for conducting CMB-based stem cell therapy.

**Table 1:** Cationic microbubble characterization

	Concentration(×10^9^/ml)	Zeta potential (mV)	Average diameter (nm)
CMB	4.28 ± 0.21	27.09 ± 3.09	1036 ± 191

### Isolation and identification of MSCs

The isolated MSCs were passaged to the third generation, and microscopic findings suggested that the MSCs were spindle-like and became more uniform after a few passages (Fig. S1A). The surface markers of MSCs were determined by flow cytometry analyses. The cells were positive for all MSC-associated markers, including CD90 and CD29, and negative for the hematopoietic stem cell markers CD45 and CD34 (Fig. S1B), confirming that the major population of adherent cells was MSCs. We treated MSCs with or without PDGF-BB (50 ng/ml) for 24 h and then detected the surface markers CD29 and CD90 to investigate whether MSCs retain the CD29- and CD44-positive phenotype after PDGF-BB treatment. PDGF-BB did not significantly alter the MSC CD29 and CD90 levels. Third-generation MSCs were used for the subsequent experiments.

### Increased MSC homing and SDF-1 expression following UTMD treatment

**Fig. 3:**
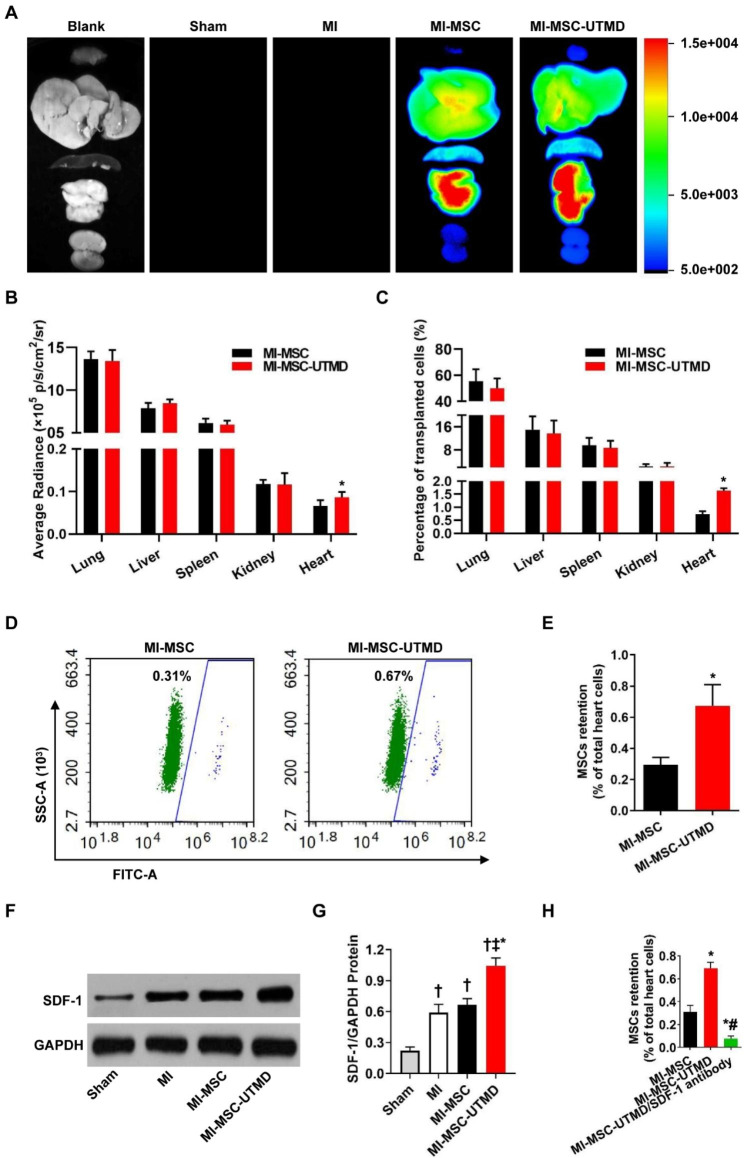
UTMD promotes the cardiac homing of MSCs by upregulating SDF-1. (**A**) Representative bioluminescence imaging of different organs harvested at 3 days after MI. (**B**) Quantification of bioluminescence signals from isolated organs of SD rats. Values are the mean ± SEM. Significant differences were determined by Student’s t test. N = 5. *p < 0.05 vs. MI-MSC. (**C**) Quantification of MSC retention in different organs in each group by PCR for the Y chromosome-specific Sry gene. Values are the mean ± SEM. Significant differences were determined by Student’s t test. N = 5. *p < 0.05 vs. MI-MSC. (**D**-**E)** Quantification of GFP-positive MSCs in whole hearts by flow cytometry. Values are the mean ± SEM. Significant differences were determined by Student’s t test. N = 5. *p < 0.05 vs. MI-MSC. (**F**-**G**) Western blotting and quantitative analysis of SDF-1 protein levels in ischemic myocardium. Values are the mean ± SEM. Significant differences were determined by using one-way ANOVA. N = 5/group. † p < 0.01 vs. Sham; ‡ p < 0.01 vs. MI; * p < 0.01 vs. MI-MSC. (**H**) Quantitative analysis of GFP-labeled MSCs in whole hearts by flow cytometry. Values are the mean ± SEM. Significant differences were determined by using one-way ANOVA. N = 5. *p < 0.01 vs. MI-MSC, # p < 0.01 vs. MI-MSC-UTMD

The hearts, livers, spleens, lungs, and kidneys were analyzed using a small animal imaging system. MSCs mainly accumulated in the lungs, livers, and spleens, the fluorescence intensity of which was significantly higher than that of the hearts. However, the quantitative analysis of the fluorescence intensity of the heart of each group showed that that of the MI-MSC-UTMD group was 1.31 times higher than that of the MI-MSC group (P < 0.05 vs. MI-MSC, Fig. 3A-B). In parallel, quantification of the distribution and homing of MSCs was also evaluated by PCR for the Y chromosome-specific Sry gene. As shown in Fig. 3C, the lungs had the highest percentage of MSC homing among the organs in all groups, and UTMD significantly increased the homing of MSCs to the infarcted myocardium compared with that in the MI-MSC group (P < 0.05 vs. MI-MSC). The proportion of GFP-positive cells in the myocardium was also assessed by flow cytometry. The number of migrating MSCs was significantly higher in the MI-MSC-UTMD group than in the MI-MSC group (P < 0.05 vs. MI-MSC, Fig. 3D-E). Next, we explored the mechanisms of UTMD-assisted homing of MSCs to the ischemic myocardium. Western blotting revealed that UTMD treatment markedly increased the expression of SDF-1 versus that in the MI and MI-MSC groups (P < 0.01, Fig. 3F-G). However, the homing of MSCs to the infarcted myocardium decreased with the addition of SDF-1 neutralizing antibody (25 µg, R&D Systems) (P < 0.01 vs. MI-MSC-UTMD, Fig. 3H & Fig. S2). These results suggest that UTMD promotes the homing of MSCs to the myocardial infarct zone by upregulating SDF-1.

### PDGF-BB increases cardiac engraftment of MSCs

**Fig. 4:**
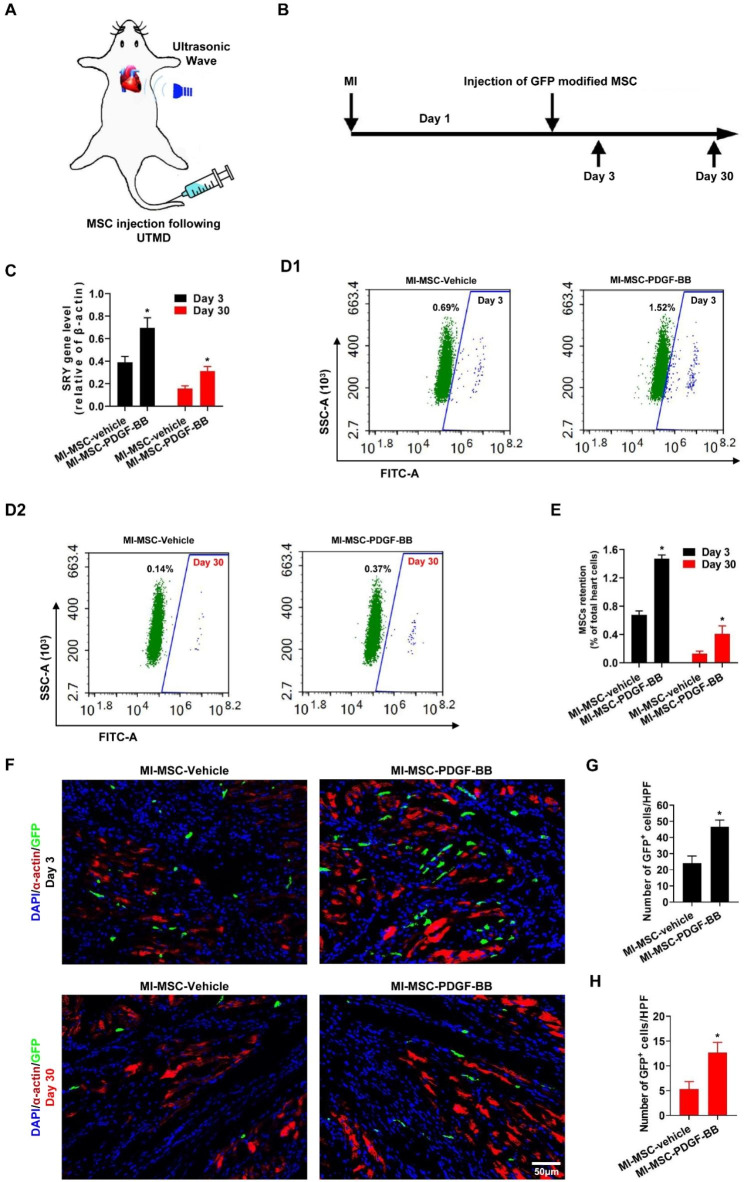
UTMD combined with PDGF-BB pretreatment further increases the cardiac engraftment of MSCs. (**A**) Schematic illustration of the transplantation of GFP-labeled MSCs mediated by UTMD. (**B**) Experimental scheme and timeline for cardiac homing studies using GFP-labeled MSCs-vehicle and MSCs-PDGF-BB. (**C**) Real-time PCR for the rat Sry gene in female rat hearts on day 3 and day 30 after MI. (**D**-**E**) Flow cytometric analysis of GFP-labeled MSCs in hearts. (F) Representative images of GFP-labeled MSCs in hearts on day 3 and day 30 after MI. Heart tissue was immunostained for α-actin (red) and DAPI (blue). Engrafted MSCs are GFP positive. Bar, 50 μm. (G-H) Quantification of MSCs in the peri-infarct area was determined by the number of GFP-positive MSCs in a representative section. Values are the mean ± SEM. Significant differences were determined by Student’s t test. N = 5/group. *p < 0.05 vs. Day MI-MSC-vehicle.

We next determined whether UTMD combined with PDGF-BB pretreatment further increased the cardiac homing of intravenously injected MSCs. Real-time PCR and flow cytometry assays were performed to detect MSCs in the heart on day 3 and day 30 after MI (Fig. 4A-B). Quantification of Sry gene expression was also evaluated using quantitative real-time PCR. As shown in Fig. 4C, Sry was detected in all MSC groups but not in the sham or MI groups. Notably, Sry expression was greatly increased in the MI-MSC-PDGF-BB group compared with that in the MI-MSC-vehicle group (P < 0.05 vs. MI-MSC-vehicle). Consistently, flow cytometric analyses showed significantly more GFP-positive cells in the ischemic myocardia of the MI-MSC-PDGF-BB group than in that of the MI-MSC-vehicle group (P < 0.05 vs. MI-MSC-vehicle, Fig. 4D-E).

The transplanted GFP-positive cells were also evaluated by fluorescence microscopy in the peri-infarct myocardial tissues on day 3 and day 30 post-MI to further examine the effect of UTMD combined with PDGF-BB pretreatment on the cardiac homing of intravenously injected MSCs. As shown in Fig. 4F-H, no GFP-positive cells were found in the sham and MI groups, and only a few GFP-positive cells were observed in the MI-MSC-vehicle group. However, many GFP-positive cells were observed in the MI-MSC-PDGF-BB group (P < 0.05 vs. MI-MSC-vehicle). Taken together, these results suggest that PDGF-BB also regulates MSC homing after delivery into ischemic myocardial tissue by UTMD.

### PDGF-BB-primed MSC transplantation by UTMD improved cardiac function after MI

**Fig. 5:**
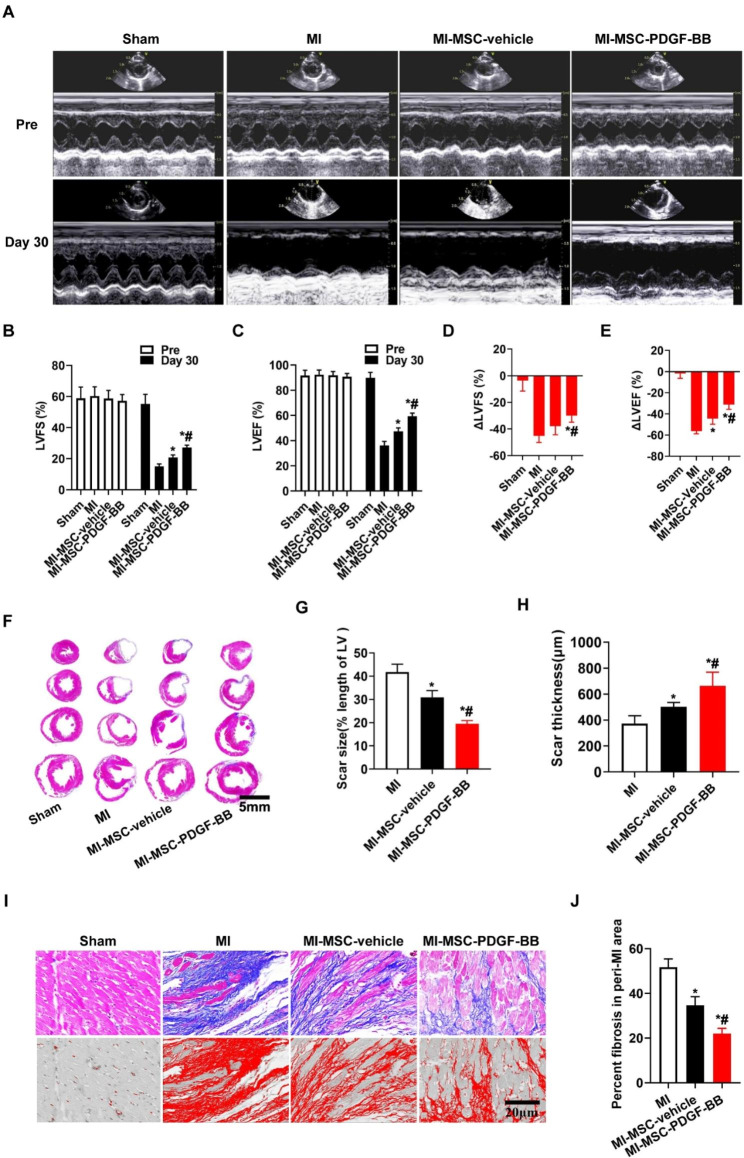
Increased cardiac function and reduced infarct size following PDGF-BB-primed MSC transplantation by UTMD. (**A**) Representative echocardiogram of rat hearts in various groups at baseline (before MI) and 30 days post-MI. (**B**-**E**) Quantification analysis of cardiac function of LVEF and LVFS. (F) Representative images of Masson’s trichrome staining of heart sections. Bar, 5 mm (G-H) Quantitative measurement of the scar size and LV wall thickness in various groups. (**I**) Masson trichrome staining at the border zone. Bar, 20 μm. (**J**) Quantitative analysis of interstitial fibrosis in perimyocardial infarction in rats among the different groups. Values are the mean ± SEM. Significant differences were determined by using one-way ANOVA. N = 5/group. *p < 0.01 vs. MI; # p < 0.01 vs. MI-MSC-vehicle

Next, we evaluated the effects of UTMD combined with PDGF-BB pretreatment on MSC therapy in a rat MI model. The heart function of rats from different groups was measured by echocardiography at baseline (before MI) and 30 days post-MI to determine the therapeutic effect of MSCs on rats post-MI. Representative images of echocardiography were taken before surgery and at 30 days after MI in rats (Fig. 5A). There was no difference in LVFS or LVEF among all groups before surgery. At 30 days post-MI, the MI group showed severely impaired LV contractile function (LVFS and LVEF) compared with that of the sham group, and the LVEF and LVFS were enhanced in all MSC-transplanted groups compared with those in the MI group. However, PDGF-BB-primed MSCs significantly improved cardiac function, as evidenced by the higher LVFS and LVEF than those in MI-MSC-vehicle group (P < 0.01 vs. MI-MSC-vehicle, Fig. 5B-E).

Fibrosis and remolding are the secondary stages after MI and can lead to a further reduction in cardiac function. Therefore, the infarct size was determined by Masson’s trichrome staining 30 days after MI. As illustrated in Fig. 5F, the normal myocardium was stained red, whereas blue indicated fibrosis tissue. The MI-MSC-vehicle group exhibited a significantly decreased infarct size compared with that of the MI group, while MI-MSC-PDGF-BB further reduced the infarct size compared to that of the MI-MSC-vehicle group (P < 0.01 vs. MI & MI-MSC-vehicle, Fig. 5G). The wall thickness of the infarct area in the MI-MSC-PDGF-BB group was thicker than that in the MI and MI-MSC-vehicle groups (P < 0.01 vs. MI & MI-MSC-vehicle, Fig. 5H). Moreover, Masson’s trichrome staining at the border zone also revealed less fibrosis but more viable tissue within the infarct region of the MI-MSC-PDGF-BB group (P < 0.01 vs. MI & MI-MSC-vehicle, Fig. 5I-J). All of these data clearly demonstrate that UTMD combined with PDGF-BB pretreatment enhances the beneficial effects of stem cell therapy and further improves cardiac function.

### PDGF-BB-primed MSCs affect cardiomyocyte apoptosis and angiogenesis post-MI

**Fig. 6:**
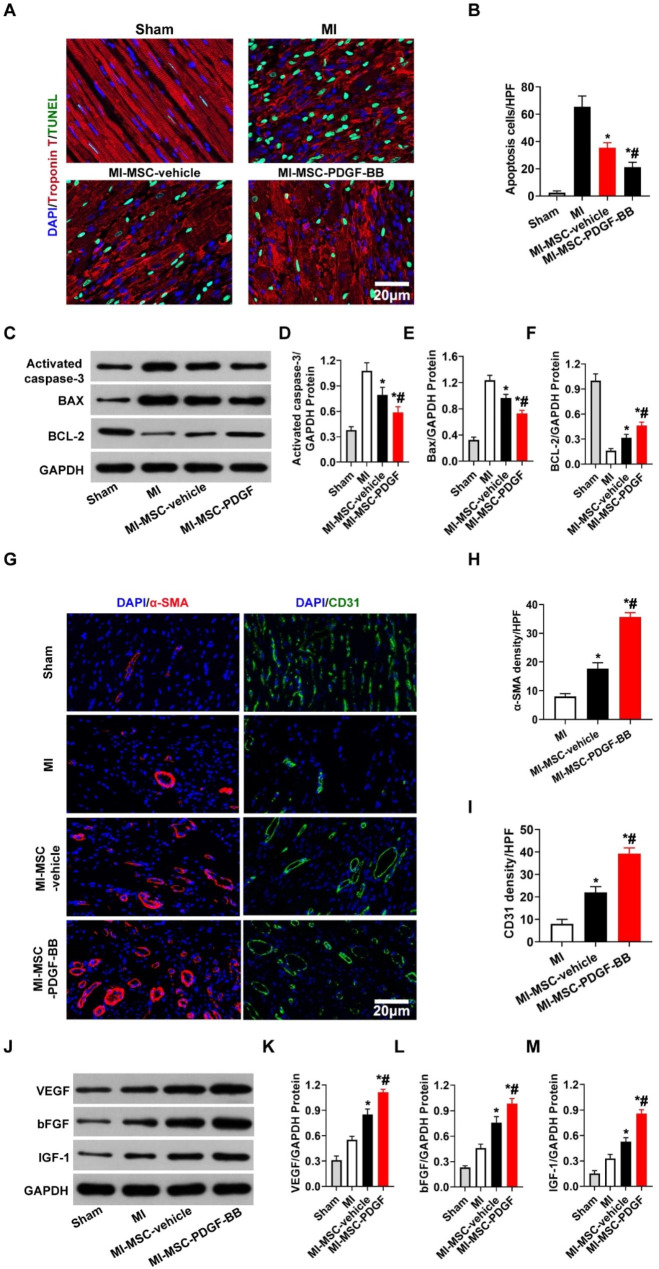
Transplantation of PDGF-BB-primed MSCs via UTMD reduces cardiomyocyte apoptosis and improves angiogenesis in rat hearts post-MI. (**A**) Representative images of TUNEL-positive cardiomyocytes in the ischemic area 30 days after MI. Apoptotic nuclei were identified as TUNEL positive (green fluorescence), and total nuclei were identified by DAPI counterstaining (blue fluorescence). Myocardium was stained using a monoclonal antibody against cardiac troponin I (red fluorescent). Bar, 20 μm. (**B**). Quantification of TUNEL-positive cardiomyocytes. (**C**-**F**) Western blotting of activated caspase 3, Bax, and BCL-2 in the ischemic heart. GAPDH was used as a loading control. (**G**) Representative images of CD31 staining and α-SMA staining in the ischemic hearts of rats 30 days post-MI. Bar, 20 μm. (**H**) Quantitative analysis of the capillary density in the ischemic heart. (I) Quantitative analysis of the arteriole density in the ischemic heart. (**J**-**M**) Protein expression of VEGF, bFGF and IGF-1 determined by Western blotting in ischemic myocardium, with GAPDH as the internal control. Values are the mean ± SEM. Significant differences were determined by using one-way ANOVA. N = 5/group. *p < 0.01 vs. MI; # p < 0.01 vs. MI-MSC-vehicle

To investigate the therapeutic mechanisms by which PDGF-BB**-**primed MSCs promote cardiac repair, we quantified cardiomyocyte apoptosis and vascular density in the infarct border area of the heart 30 days post MI. The MI-MSC-PDGF-BB group demonstrated fewer apoptotic cardiomyocytes in the ischemic border zone than the MI or MI-MSC-vehicle group (P < 0.01 vs. MI & MI-MSC-vehicle, Fig. 6A-B). To further confirm the effect of PDGF-BB**-**primed MSCs on cardiomyocyte apoptosis, the expression of apoptosis-related proteins was detected by Western blotting, which indicated that the expression of Bax and cleaved-Caspase 3 was obviously decreased and the expression of BCL-2 was increased in the MI-MSC-PDGF-BB group versus the MI-MSC-vehicle and MI groups (P < 0.01 vs. MI & MI-MSC-vehicle, Fig. 6C-F).

Angiogenesis and arterialization were evaluated by immunofluorescence staining of CD31 and αSMA in the peri-infarcted area. The arteriole density was significantly increased in the MI-MSC-PDGF-BB group compared to that in the MI-MSC-vehicle and MI groups 30 days after MI (P < 0.01 vs. MI & MI-MSC-vehicle, Fig. 6G-I). Similar trends were observed in capillary density (P < 0.01 vs. MI & MI-MSC-vehicle, Fig. 6G-I). Furthermore, the expression of angiogenic factors in ischemic cardiomyopathic hearts was evaluated by Western blotting. The expression levels of VEGF, bFGF, and IGF-1 in ischemic cardiomyopathic hearts were significantly higher in the MI-MSC-PDGF-BB group than those in the MI-MSC-vehicle and MI groups (P < 0.01 vs. MI & MI-MSC-vehicle, Fig. 6J-M).

### PDGF-BB priming increases the cardioprotective effects of MSCs

We examined whether conditioned medium (CM) from PDGF-BB**-**primed MSCs affects the survival of the cardiomyocyte cell line H9c2. H9c2 cells were treated with 200 µM H_2_O_2_ for 6 h. The flow cytometry results indicated that vehicle-MSC-CM decreased the apoptosis of H9c2 cells, and compared with vehicle-MSC-CM, PDGF-BB-MSC-CM further reduced the apoptosis of H9c2 cells (all P < 0.01, Fig. S3).

### PDGF-BB promotes MSC migration and protects MSCs against apoptosis

**Fig. 7:**
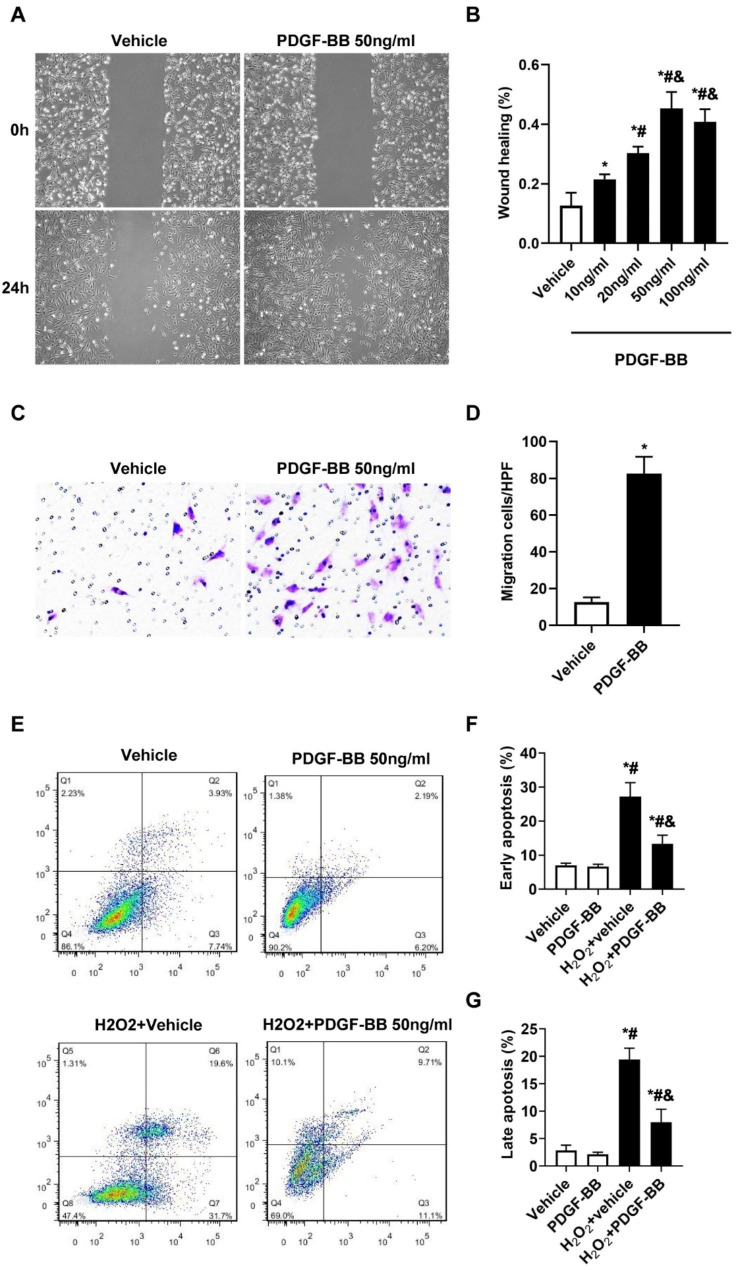
PDGF-BB promotes MSC migration and protects MSCs against apoptosis. (**A**-**B**). The wound healing assay illustrated MSC migration 24 h after PDGF-BB treatment at different concentrations. Bar, 200 μm. N = 6/group. *p < 0.01 vs. vehicle; # p < 0.01 vs. 10 ng/ml; & p < 0.01 vs. 20 ng/ml. (**C**-**D**). Cell migration was detected by Transwell assay after 24 h of treatment with or without PDGF-BB. Bar, 100 μm. N = 6/group. *p < 0.05 vs. vehicle. (**E**-**G**). MSC apoptosis was assessed by Annexin V/PI staining followed by flow cytometry analysis. H_2_O_2_, 200 µM, 6 h. Values are the mean ± SEM. Significant differences were determined by using one-way ANOVA. N = 6/group. *p < 0.01 vs. vehicle; # p < 0.01 vs. PDGF-BB; & p < 0.01 vs. H_2_O_2_-vehicle

MSC migration and survival in ischemic environments are crucial for their therapeutic efficacy in ischemic diseases. Hence, we explored the effects of PDGF-BB on the migration and anti-apoptosis of MSCs in vitro. We treated MSCs with different concentrations (0, 10, 20, 50 and 100 ng/ml) of PDGF-BB for 24 h. Wound healing assays revealed that PDGF-BB promoted MSC migration in a dose-dependent manner, with the maximum effect observed at a concentration of 50 ng/ml PDGF-BB (all P < 0.01, Fig. 7A-B). The effects of the optimal dose of 50 ng/ml PDGF-BB on MSC migration were further evaluated by Transwell assays. The results indicated that an optimal dose of 50 ng/ml PDGF-BB significantly stimulated the migration of MSCs (P<0.01 vs. vehicle. Figure 7 C-D). These findings are consistent with those of previous studies showing that PDGF-BB chemoattracts several cell types, including MSCs [12, 16, 27–29]. As hypoxia and oxidative stress in the ischemic area of the post-MI heart are believed to be the main causes of the death of transplanted MSCs [30–32], we induced oxidative stress in vitro using H_2_O_2_. MSC apoptosis was measured by annexin V-FITC/PI double-staining flow cytometry. As shown in Fig. 7E-G, H_2_O_2_ exposure increased the number of MSCs in quadrants 2 and 3 (Q2 and Q3) compared to the vehicle group (P<0.01), suggesting that H_2_O_2_ induced early and late apoptosis. When MSCs were coincubated with 50 ng/ml PDGF-BB for 24 h, the percentage of apoptotic cells significantly decreased (P < 0.01 vs. H_2_O_2_-vehicle.). Taken together, these findings suggest that PDGF-BB induces the migratory and anti-apoptotic properties of MSCs.

### The PI3K/Akt pathway is responsible for the PDGF-BB-induced migration and anti-apoptosis of MSCs

**Fig. 8:**
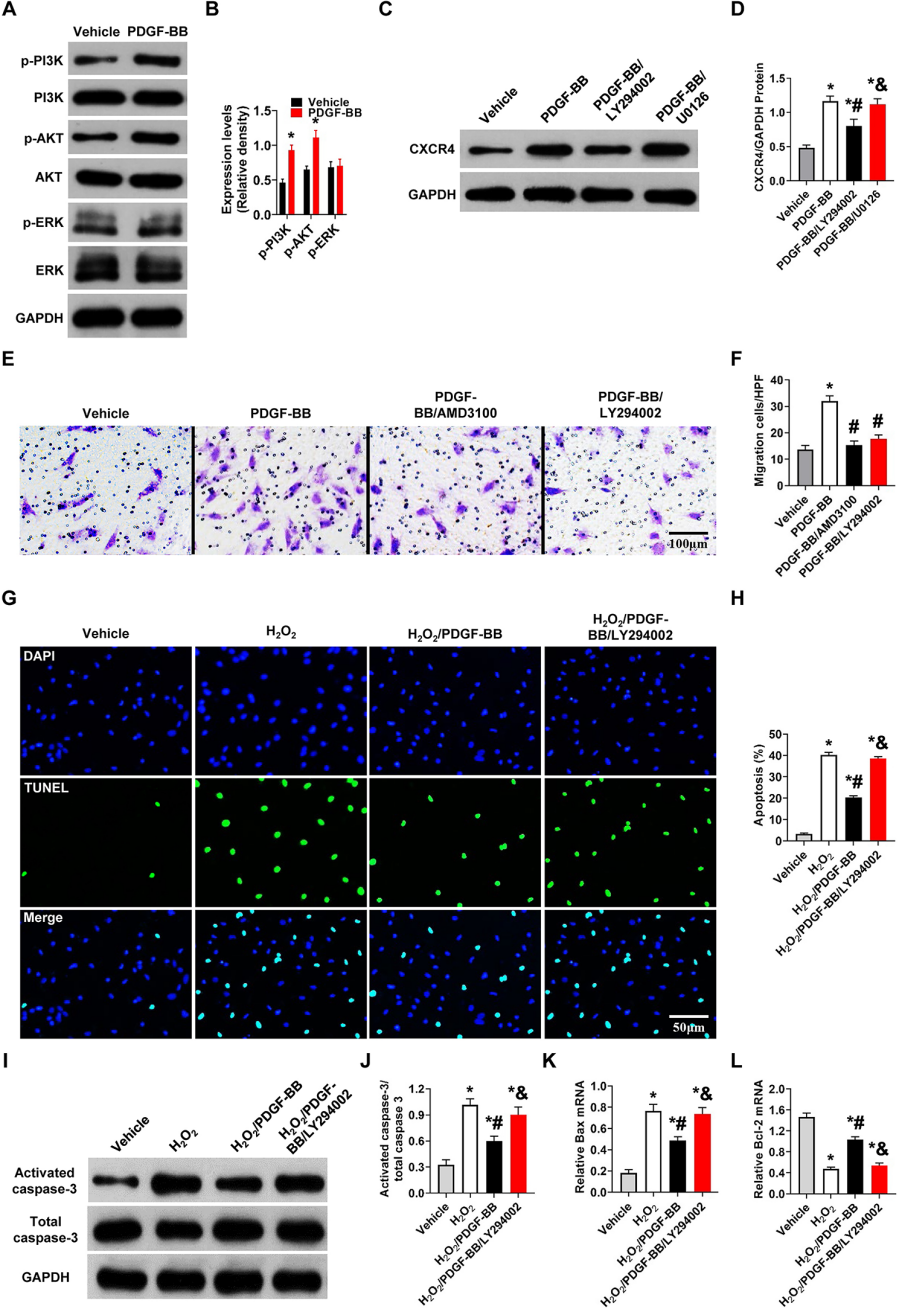
PDGF-BB promotes MSC migration and protects MSCs against apoptosis via PI3K/Akt signaling. (**A**-**B**) Western blot analysis of total and phosphorylated PI3K, Akt, and ERK in MSCs with or without PDGF-BB treatment. PDGF-BB, 50 ng/ml. Values are the mean ± SEM. Significant differences were determined by Student’s t test. N = 6/group. *P < 0.05 vs. vehicle. (**C**-**D**) Western blot analysis of CXCR4 in MSCs. LY294002, 10 µM, U0126, 10 µM, 2 h before PDGF-BB treatment. Values are the mean ± SEM. Significant differences were determined by using one-way ANOVA. N = 6/group. *p < 0.01 vs. vehicle; # p < 0.01 vs. PDGF-BB; & p < 0.01 vs. PDGF-BB/LY294002. (**E**-**F**). MSC migratory capacities were evaluated by Transwell assay. LY294002, 50 µM. AMD3100, 44 nM. PDGF-BB, 50 ng/ml. Bar, 100 μm. Values are the mean ± SEM. Significant differences were determined by using one-way ANOVA. N = 6/group. *p < 0.01 vs. vehicle; # p < 0.01 vs. PDGF-BB; & p < 0.01 vs. PDGF-BB/AMD3100. (**G**-**H**). MSC apoptosis was evaluated by TUNEL assay. Bar, 50 μm. (**I**-**J**) Western blot analysis of activated caspase-3 in MSCs. (**K**-**L**) Bax and BCL-2 mRNA expression levels were determined by RT‒PCR in MSCs. H_2_O_2_, 200 µM, 6 h. LY294002, 30 µM. Values are the mean ± SEM. Significant differences were determined by using one-way ANOVA. N = 6/group. *p < 0.01 vs. vehicle; # p < 0.01 vs. H_2_O_2_; & p < 0.01 vs. H_2_O_2_/PDGF-BB

Given the critical role of PI3K/Akt and ERK1/2 in the downstream signaling of PDGF-BB, we quantified the phosphorylation levels of PI3K, Akt and ERK1/2 in the presence or absence of PDGF-BB. As shown in Fig. 8A-B, PDGF-BB significantly increased the phosphorylation levels of PI3K and Akt (P<0.01 vs. vehicle) but not ERK1/2. The PI3K/Akt signaling pathway has been observed to regulate the secretion of CXCR4 [33, 34]. Indeed, the present study also showed that PDGF-BB increased CXCR4 protein expression, which was significantly reduced by treatment with LY294002 (P<0.01 vs. PDGF-BB, Fig. 8C-D). However, the pretreatment of MSCs with U0126 did not reduce PDGF-BB-induced CXCR4 protein expression (Fig. 8C-D). Thus, we tested whether PI3K/Akt and CXCR4 play a role in the PDGF-BB-induced migration of MSCs. LY294002 and AMD3100 were used to inhibit the activation of PI3K/Akt and CXCR4. As shown in Fig. 8E-F, the migration of MSCs toward SDF-1 was mostly blocked, and the number of migrating MSCs significantly decreased with the addition of LY294002 or AMD3100 (P<0.01 vs. PDGF-BB). These findings indicate that PDGF-BB-mediates PI3K/Akt, and CXCR4 activation correlates with the PDGF-BB-induced migration of MSCs.

The PI3K/Akt pathway is also involved in the transduction of antiapoptotic signals in various cells under oxidative injury [35–38]. Thus, we speculated that the capability of PDGF-BB to prevent MSCs from undergoing apoptosis was attributable to PI3K/Akt activation. As shown in Fig. 8G- H, H_2_O_2_-induced MSC apoptosis increased the number of TUNEL + cells, and few TUNEL + cells were present in the H_2_O_2_/PDGF-BB group (P<0.01 vs. H_2_O_2_). However, pretreatment of MSCs with LY294002 significantly abolished the protective effects of PDGF-BB against MSC apoptosis. To confirm the mechanism of this protective effect, we examined the expression of apoptosis-related genes in PDGF-primed MSCs in the presence or absence of LY294002 after H_2_O_2_ treatment. Our data demonstrated that compared with H_2_O_2_, PDGF-BB treatment resulted in a significant decrease in caspase-3 activity, which was inhibited by LY294002 (P<0.01, Fig. 8I-J). The same results were obtained for Bax and BCL-2 expression (P<0.01, Fig. 8K-L). These results indicate that PI3K/Akt is partly responsible for the anti-apoptotic role of PDGF-BB in H_2_O_2_-induced MSC death.

## Discussion

In this study, we employed a noninvasive method, targeted delivery of PDGF-BB-primed MSCs to the rat heart using UTMD, to investigate the ability of PDGF-BB-primed MSCs to improve tissue repair after MI and determine the mechanism(s) underlying the improved retention and reparative effects of PDGF-BB-primed MSCs. We present evidence that UTMD enhances the targeted delivery of MSCs into the infarcted myocardium following upregulation of SDF-1 expression. We observed that myocardium-targeted delivery of MSCs by UTMD reduces myocardial apoptosis and infarct size, increases vascular density and improves cardiac function. However, intravenous infusion of PDGF-BB-primed MSCs following UTMD had a more prominent ability to protect against cardiac injury. In addition, we identified that PDGF-BB pretreatment modulates the migration and survival of MSCs via the PI3K/Akt pathway and CXCR4 activation. Thus, our study indicates that UTMD combined with PDGF-BB pretreatment can further enhance the therapeutic potential of MSCs for ischemic heart diseases.

Stem cell transplantation is a promising therapeutic strategy for ischemic heart diseases. However, MSCs do not efficiently home to the infarcted areas, limiting the improvement of cardiac function [39]. Ultrasound combined with microbubbles as a novel noninvasive system for the delivery of drugs or genes [19, 40, 41] could help improve the homing ability and transplantation efficiency of MSCs following AMI [7, 8, 42]. However, the mechanisms of MSC-targeted delivery into the infarcted myocardium remain unclear. Some studies have shown that ruptured capillaries and widened endothelial gaps caused by UTMD promote the transmigration of MSCs from the blood vessels to the ischemic region [8, 42, 43]. Others have demonstrated that UTMD can change the microenvironment within ischemic myocardial tissue caused by the local inflammatory response and enhance the adhesion of transplanted MSCs to endothelial cells, improving MSC homing [7]. In this study, the possible mechanism of MSC-targeted delivery into the infarcted myocardium was also investigated. We demonstrated that UTMD promotes the migration of transplanted MSCs to the ischemic region by upregulating SDF-1. Many studies have shown that the CXCR4/SDF-1 axis is a prerequisite for stem cell homing [44, 45]. CXCR4, a chemotactic receptor recognizing the chemokine SDF-1, has been found to be expressed on the surface of MSCs and can induce the site-directed homing of MSCs along a gradient of chemokine concentrations when activated by SDF-1 [46]. Thus, the concentration of SDF-1 correlates well with the therapeutic effects of stem cell transplantation. This study demonstrated higher levels of SDF-1 expression in the MI-MSC-UTMD group than in the other groups, and the homing of MSCs to the infarcted myocardium decreased with the addition of SDF-1-neutralizing antibody. Thus, UTMD can boost SDF-1 expression at sites of injury, triggering MSC migration toward the injured area. The UTMD-mediated upregulation of SDF-1 expression in the ischemic myocardium may be induced by its biological effects generated by the destruction of MBs with US irradiation. In addition, the HUVEC targeting capability of CMBs was estimated by microscopy. Due to electrostatic interactions, the attachment of CMBs to HUVECs was obviously higher than that of NMBs, which may enhance cavitation effects and subsequently SDF-1 expression and MSC migration. Previous studies have demonstrated that ultrasonic irradiation combined with MBs can cause myocardial cell damage and other adverse reactions [47], indicating that the irradiation parameters for MSC transplantation by UTMD require optimization. We previously reported that ultrasound with a low intensity of 2 W/cm^2^ and a frequency of 1 MHz did not adversely affect cell viability [19]. Therefore, we adopted identical ultrasonic parameters in this study and demonstrated that irradiation parameters with an acoustic intensity of 2.0 W/cm^2^ and frequency of 1 MHz did not noticeably impact myocardial apoptosis in vivo, even with MBs.

However, MSC transplantation relying only on UTMD has limitations because MSCs progressively downregulate CXCR4 expression after ex vivo expansion [48, 49] and lose their ability to migrate toward SDF-1 [9, 10], suggesting that increasing CXCR4 expression might be an important strategy to improve the migration of MSCs and accelerate the recovery of injured tissues. PDGF-BB is a powerful chemotactic factor for mesenchymal lineage cells [50–52]. Whether pretreatment with PDGF-BB can promote the migration of MSCs via the upregulation of CXCR4 has yet to be examined. In the present study, we found that PDGF-BB pretreatment increased the expression of CXCR4 and enhanced the migration of MSCs toward SDF-1 in vitro. However, MSCs were preincubated with AMD3100, resulting in a significant decrease in the migration of PDGF-BB-treated MSCs, which was matched with the downregulation of CXCR4 expression. These results suggest involvement of the CXCR4/SDF-1α axis in PDGF-BB-induced MSC migration. A previous study demonstrated that the PI3K/Akt and ERK signaling pathways primarily mediate PDGF-BB-induced signaling. However, the effects of interactions between these signaling pathways in MSCs remain poorly understood. PI3K regulates the phosphorylation of AKT, and the subsequent PI3K/AKT downstream signaling pathway has been suggested to play a regulatory role in numerous cell proliferation and activation processes [12, 13]. In contrast, the ERK signaling pathway has been reported to play a key role in transducing signals from cell-surface receptors to the nucleus, thereby regulating the activation and proliferation of cells [53, 54]. Therefore, the phosphorylation of PI3K, AKT and ERK1/2 was also examined in the present study. Our results indicate that PDGF-BB upregulates the expression of phosphorylated PI3K and Akt but not ERK1/2. Moreover, the data also demonstrated that the increases in CXCR4 and migration were abolished by the addition of a chemical inhibitor of LY294002 prior to PDGF-BB treatment. Collectively, these data indicate a role for the PI3K/Akt signaling pathway in PDGF-BB-induced MSC migration.

Although the increased migration response of MSCs may contribute to higher transplantation efficiency in clinical applications, the hostile environment of injured heart tissue, including hypoxia and oxidative stress, causes excessive cell death, leading to an urgent need to enhance the resistance of MSCs to apoptosis [30–32]. Consequently, improved survival of MSCs in oxidative injury is necessary. Therefore, we explored the antiapoptotic effect of PDGF-BB on MSCs under oxidative stress induced by H_2_O_2_ and revealed that PDGF-BB could significantly reduce H_2_O_2_-induced MSC apoptosis at both the early and late stages and enhance their survival when transplanted into the hostile environment of a post-MI heart. In addition, PDGF-BB-primed MSCs exhibited a cytoprotective effect by inhibiting cell apoptosis of the H9c2 cardiomyocyte cell line and myocardial cell apoptosis in a rat model with post-MI heart failure. All the aforementioned results suggest that the effects of PDGF-BB-primed MSCs are mainly due to a paracrine effect and not myocardial differentiation. The PI3K/AKT pathway has been identified as an antiapoptotic effector of PDGF-BB signaling [55, 56]. Thus, we examined the relationship between the antiapoptotic effects of PDGF-BB preconditioning in MSCs and the activation of PI3K/AKT signaling. We then employed LY294002 to inhibit the corresponding kinases. The results demonstrated that PDGF-BB treatment activated the PI3K/AKT signaling pathway to resist H2O2-mediated activation of caspase 3 expression and cell apoptosis. Taken together, these results indicate that the PI3K/Akt pathway contributes to both the enhanced migration and the antiapoptotic effect of PDGF-BB on MSCs.

The present study demonstrated that UTMD facilitated the homing of MSCs to the MI area by upregulating SDF-1, and PDGF-BB pretreatment protected MSCs against apoptosis and enhanced their ability to migrate toward SDF-1. However, whether pretreatment with PDGF-BB combined with UTMD has additional effects of MSCs on post-MI cardiac remodeling is unclear. We pretreated MSCs with PDGF-BB and transplanted them into the infarcted area of the heart by UTMD in a rat model of MI and examined the efficacy of different treatments for myocardial infarction via echocardiography, bioluminescent imaging, and pathological analysis. Our in vivo studies showed that UTMD combined with PDGF-BB pretreatment significantly promotes the cardiac homing and cardioprotection of MSCs. We demonstrated that PDGF pretreatment combined with UTMD markedly increases the cardiac homing of intravenously injected MSCs in post-MI rats by tracking MSCs with DiR or genetic GFP labeling. By echocardiography, we found that postinfarction left ventricular function improved most in the MI-MSC-PDGF-BB group versus the MI-MSC-vehicle group. The pathological examination indicated that PDGF-BB-treated MSC transplantation mediated by UTMD notably increased the capillary density and reduced cardiomyocyte apoptosis and fibrosis in the peri-infarct area. Based on the current findings, we proposed several mechanisms to account for the efficacy of UTMD combined with PDGF-BB pretreatment from two aspects: improved cell migration and survival and enhanced paracrine functions. First, as shown above, PDGF-BB pretreatment increased the cell surface expression of CXCR4, and UTMD upregulated the expression of SDF-1 in myocardial infarct tissue, while the interaction between CXCR4 and SDF-1 improved the migration and homing abilities of implanted MSCs. In addition, preconditioning with PDGF-BB increased implanted cell survival, as evidenced by the above comments. Thus, compared with UTMD alone, UTMD combined with PDGF-BB pretreatment resulted in greater numbers of homing MSCs, which is essential for the success of stem cell therapy. Second, as demonstrated in previous studies, stem cells rarely differentiate into cardiomyocytes, and paracrine effects are heavily implicated in MSC-based improvements in cardiac dysfunction after MI [57, 58]. One previous study indicated that MSCs secrete various bioactive factors [59, 60]. In the current study, our results showed that, compared with UTMD alone, UTMD combined with PDGF-BB pretreatment further enhanced the paracrine proangiogenic properties of MSCs, as evidenced by increased expression of proangiogenic cytokines, such as VEGF, bFGF, and IGF-1, and elevated capillary density in vivo, which led to decreased myocardial apoptosis, reduced scar area and improved cardiac function.

Although this study had some promising results, there are several limitations to be considered. First, although UTMD may improve the homing of stem cells in the infarct area and around the infarct area after MI, many researchers have reported that the application of ultrasound and microbubbles could cause adverse bioeffects. Safer and more effective parameters should be explored and optimized before clinical trials begin. Second, MSC transplantation mediated by UTMD involves multiple mechanisms and various cytokines. This study on the mechanism of MSC migration and homing promoted by UTMD mainly focuses on the increase in SDF-1 expression in the microenvironment of ischemic myocardial tissue. Other cytokines may be helpful for the homing of stem cells, which requires further study. Third, this is a preclinical study of UTMD technology for cellular therapy, and there remains considerable room for future development.

## Conclusions

In conclusion, the present study demonstrated that UTMD combined with PDGF-BB pretreatment increases the therapeutic effect of grafted cells by improving MSC migration and survival, reducing cardiomyocyte apoptosis, decreasing fibrosis, increasing microvessel density and improving cardiac function. The combination of UTMD and PDGF-BB pretreatment represents an emerging potential approach to cellular therapy for AMI.

### Electronic supplementary material

Below is the link to the electronic supplementary material.

Supplementary Material 1

## Data Availability

The data supporting the findings of this study are available from the corresponding author upon reasonable request.

## Abbreviations

AMI: Acute myocardial infarction
bFGF: Basic fibroblast growth factor
CMB: Cationic microbubbles
CXCR4: CXC-chemokine receptor 4
ERK: Extracellular signal-regulated kinase
FACS: Flow cytometry analysis
HUVECs: Human umbilical vein endothelial cells
IGF-1: Insulin-like growth factor-1
GFP: Green fluorescent protein
LVEF: Left ventricular ejection fraction
LVFS: Left ventricular fractional shortening
MSCs: mesenchymal stem cells
NMB: Neutral microbubbles
PDGF-BB: Platelet-derived growth factor-BB
PI3K: Phosphoinositide 3-kinase
SDF-1: Stromal cell-derived factor 1
SEM: Scanning electronic microscopy
UTMD: ultrasound-targeted microbubble destruction
VEGF: Vascular endothelial growth factor
