# Supplementary material for: Ultrasound-targeted microbubble destruction promotes PDGF-primed bone mesenchymal stem cell transplantation for myocardial protection in acute Myocardial Infarction in rats
Source: J Nanobiotechnology. 2023 Dec 15;21:481. doi: 10.1186/s12951-023-02204-7 (PMC10725038; doi:10.1186/s12951-023-02204-7)
Supplement: Supplementary file 1 — Supplementary Material 1 [file 12951_2023_2204_MOESM1_ESM.docx]

**Ultrasound-Targeted Microbubble Destruction Promotes** **PDGF-Primed Bone** **Mesenchymal Stem Cells Transplantation for Myocardial Protection in** **Acute** **Myocardial Infarction of rats**

Zhenxing Sun^a,b,c,d^, Yu Cai^c,d^, Yihan Chen^c,d^, Qiaofeng Jin^c,d^, Ziming Zhang^c,d^, Li Zhang^c,d^, Yuman Li^c,d^, Lei Huang^c,d^, Jing Wang^c,d^, Yali Yang^c,d^, Qing Lv^c,d^, Zhengyang Han^c,d,e^ †, Mingxing Xie^c,d^†, Xiangming Zhu^a,b^ †

1. School of Continuing Education, Anhui Medical University, Hefei 230031, China
2. Department of Ultrasound, The First Affiliated Hospital of Wannan Medical College, Wuhu 241001, Anhui, China
3. Department of Ultrasound medicine, Union Hospital, Tongji Medical College, Huazhong University of Science and Technology, Wuhan 430022, China
4. Hubei Province Key Laboratory of Molecular Imaging, Wuhan 430022, China
5. Department of Ultrasound, The First Affiliated Hospital of Zhengzhou University, Zhengzhou 450099, Henan, China

† Co-senior author

**Corresponding address:**

Xiangming Zhu, No.2 Zheshan west Road, Wuhu City 241001, Anhui province, China. Tel: 0553-5738279. Email: [zhuxmwuhu@163.com](mailto:zhuxmwuhu@163.com)

Mingxing Xie, No. 1277 Jiefang Ave, Wuhan 430022, China. Tel: 86-27-85726430. E-mail address: xiemx@hust.edu.cn

Zhengyang Han, No.1 Longhu Zhonghuan Road, Jinshui District, Zhengzhou 430022, Henan. Tel: 0371-66913114. E-mail address: 1415034454@qq.com


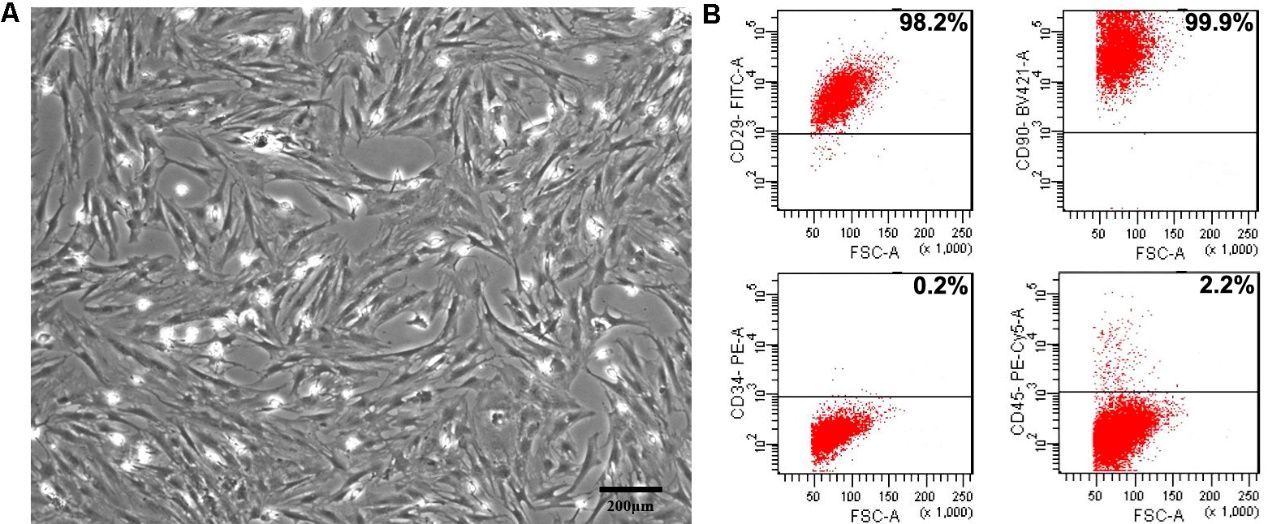


**Figure S1. Characterization of MSCs.**

A. The morphology observation of MSCs under the optical microscopy. Bar, 200μm; B. Flow cytometry of MSCs for mesenchymal CD29, 90, 34, and 45 markers. Unstained cells were used as control.


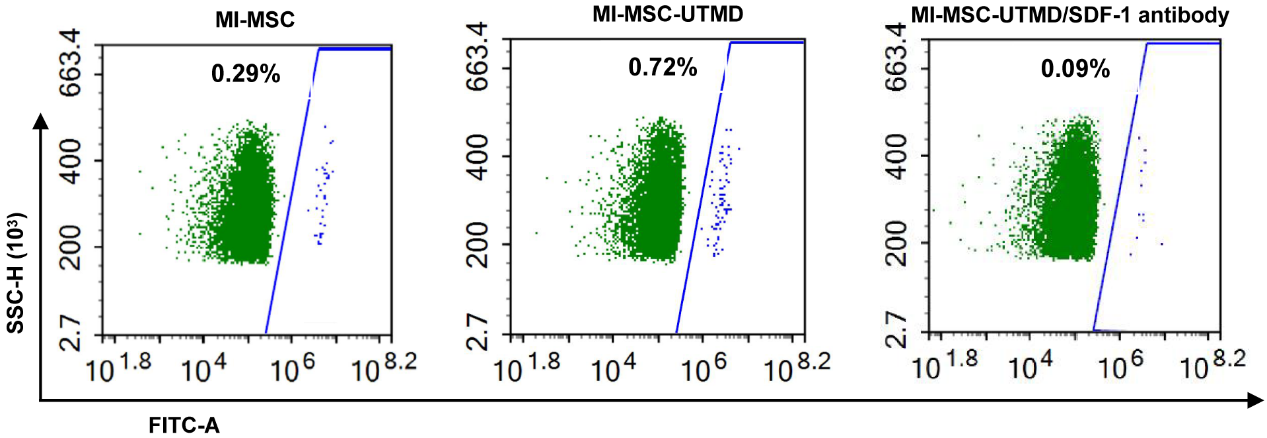


**Figure S2 Representative flow cytometry images of GFP-labeled MSCs in hearts**


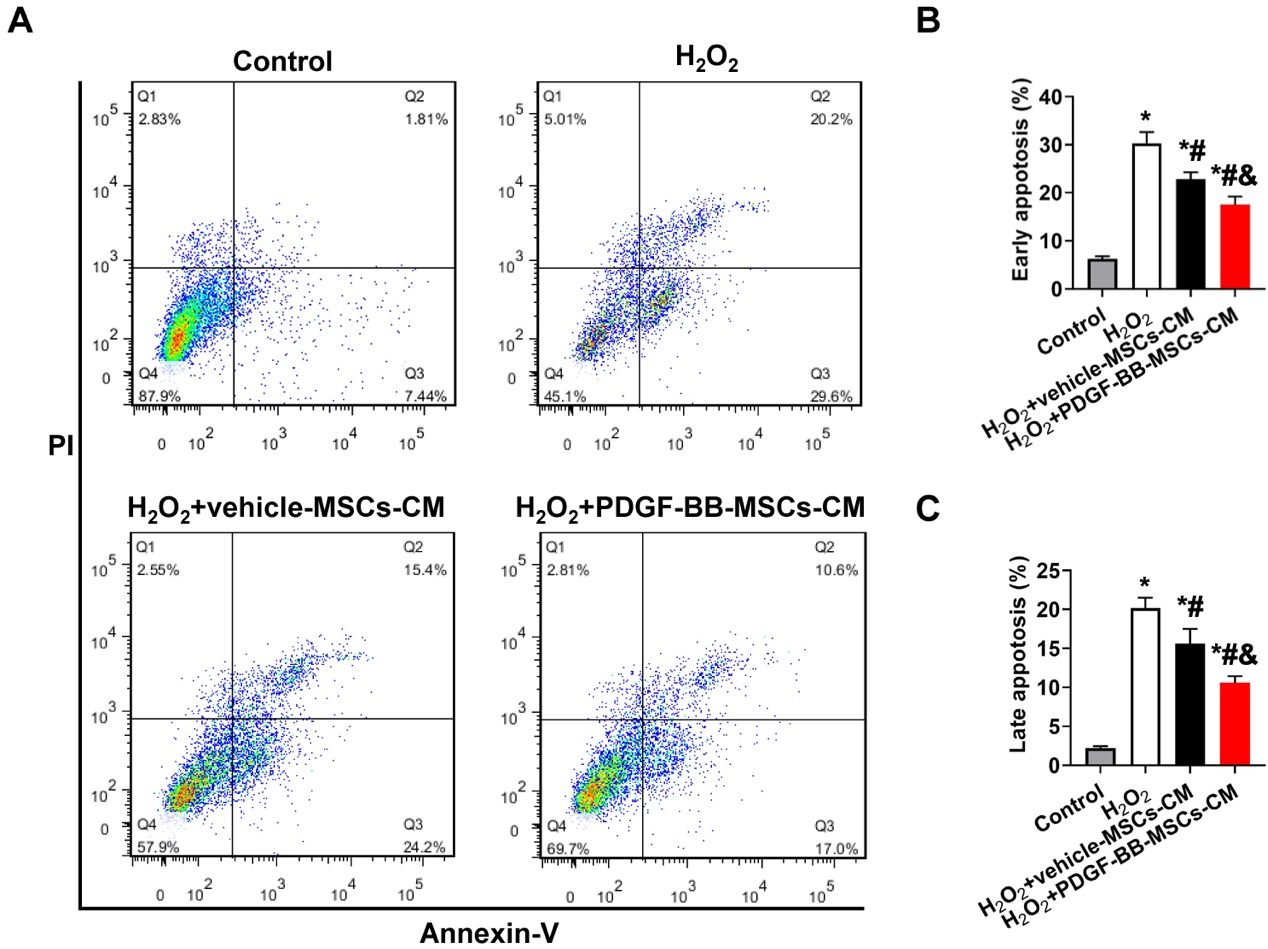


**Figure S3 Anti-apoptotic effect of CM from PDGF-BB-primed MSC on H9C2 myocardial cells subjected to H_2_O_2_.**

(A-C) H9c2 apoptosis was assessed by Annexin V/PI staining followed by flow cytometry analysis. H_2_O_2_, 200 µM, 6 hours. N=6. *p<0.01 vs. Control; # p<0.01 vs. H_2_O_2_; & p<0.01 vs.H_2_O_2_-vehicle-MSCs-CM.
